# Incidentalomas: managing risks

**DOI:** 10.1590/0100-3984.2015.48.4e3

**Published:** 2015

**Authors:** Rubens Chojniak

**Affiliations:** 1 Director, Imaging Department, A.C.Camargo Cancer Center, Professor at School of Medicine, Universidade Nove de Julho (Uninove), São Paulo, SP, Brazil. E-mail: chojniak@accamargo.org.br.

Incidentaloma is the medical term for incidentally found asymptomatic tumors. Such imaging findings have been increasingly frequent as the use of sectional imaging methods is disseminated^(1)^. Its incidence can hardly be established, but it is known that pulmonary nodules may be identified at up to 50% of chest computed tomography (CT) studies in smoking individuals and at up to 25% of studies of non-smokers^(2,3)^. At CT colonography, incidental findings are identified in up to 70% of patients and, at abdominal CT, renal and hepatic findings occur in about 15% of the individuals^(3-5)^. Also, asymptomatic nodules are identified in up to 67% of individuals submitted to thyroid ultrasonography^(3)^.

Incidental imaging findings such as aortic aneurysms may be clinically significant, determining interventions that may change the natural course of the disease. However, most incidental findings constitute a form of overdiagnosis. Overdiagnosis is the diagnosis of a disease that never will cause symptoms or death of the patient, and is nowadays considered to be a public health problem^(3,6)^. Usually, such diagnoses refer either to lesions with a benign nature or to least aggressive malignant lesions. Some cancers do not progress or are so indolent that will never produce symptoms in the patients who will die for other causes^(6)^.

The uncertainty and anxiety may lead to the automatic adoption of investigative measures with the objective of identifying the nature of any lesion that might even remotely represent a cancer^(7)^. Such investigations, even imbued with the best intentions, may cause morbidity and even death in individuals who have harmless lesions^(7)^.

A pediatric neurosurgeon has defined a new acronym to describe the risks associated with incidental imaging findings: VOMIT

– *victims of modern imaging technologies*^(8)^.

In a letter to the Editor published in the **Radiology** journal, a radiologist, Dr. William J. Casarella, reported his personal experience with incidentalomas, starting with a virtual colonoscopy indicated in his routine check-up, that did not revealed any finding in his bowels. However, other findings triggered further investigations, percutaneous biopsy, videothoracoscopy, requiring some days in a hospital and a few weeks for him to get recovered. The experience has led Dr. Casarella to call his peers’ attention to the potential risks caused by incidental findings^(9)^.

The management of incidental findings represents a clinical dilemma, but it is also influenced by other factors, such as cultural issues, the people’s belief that “you should better be safe than sorry”. The reflexive adoption of such an assumption might lead to irremediable injuries caused by excessive investigation of indolent lesions^(7,10)^. Another factor influencing the management of incidentalomas would be the defensive medicine practice. The fear of “missing” a cancer and to become legally liable may affect the physician’s decisions, leading to a greater number of investigative procedures. The society seems to be more attentive to the shortage than to the excess of care and intervention^(7,10)^. Certainly, the role played by economic interests of medical equipment companies, hospitals and physicians, besides the health services remuneration model should not be underestimated^(10)^.

Then, what could we do in face of this scenario? Considering that incidentalomas are eminently radiological entities, the radiologist’s opinion about an incidental imaging finding plays a critical role in the subsequent decision making process and may strongly interfere in such a scenario. Initially, as we have always done, we should know everything about this entity, namely, the frequency of occurrence of such findings, the spectrum of possible diagnoses, their respective rates of prevalence, radiological appearances and biological behavior^(10,11)^. More experienced radiologists or subspecialists dedicated to a specific field tend to indicate a lower number of additional examinations and tests to investigate incidental findings^(12-14)^.

Consistency is also a way to enhance the medical community’s confidence in radiological results. The rates of recommendation for additional imaging studies are highly variable amongst the institutions and also amongst radiologists working in a same institution^(12-15)^. The adoption of investigation guidelines levels up the practice standards, offering greater confidence when the no further investigation is reccomended^(12)^. Several authors and medical societies pursue standardization initiatives in relation to the management of incidentalomas. One can mention the Fleischner Society’s guidelines for asymptomatic pulmonary nodules detected at CT^(16)^, and the American College of Radiology (ACR) initiative that has organized a committee for incidental findings, and published management guidelines for the majority of incidental abdominal findings. Also, the ACR recommends that flowcharts are made widely available for radiologists during the preparations of their imaging reports^(17)^.

Finally, we could bring the “better safe than sorry” concept into question whenever there is a situation where the risk for missing a cancer is smaller than that caused by the investigation of ordinary benign and indolent lesions.

Therefore, one should give an opinion including the risk/benefit concept, in order to avoid causing harm to those who are healthy.
